# m^6^A Modification of Long Non-Coding RNA HNF1A-AS1 Facilitates Cell Cycle Progression in Colorectal Cancer via IGF2BP2-Mediated CCND1 mRNA Stabilization

**DOI:** 10.3390/cells11193008

**Published:** 2022-09-27

**Authors:** Yibo Bian, Yang Wang, Shufen Xu, Zhishuang Gao, Chao Li, Zongyao Fan, Jie Ding, Keming Wang

**Affiliations:** 1 Department of Oncology, Second Affiliated Hospital of Nanjing Medical University, Nanjing 210029, China; 2 Department of General Surgery, The First Affiliated Hospital of Nanjing Medical University, Nanjing 210029, China; 3 Department of Urology, Second Affiliated Hospital of Nanjing Medical University, Nanjing 210029, China

**Keywords:** colorectal cancer, m^6^A, HNF1A-AS1, IGF2BP2, CCND1

## Abstract

Background: Long non-coding RNAs modulate tumor occurrence through different molecular mechanisms. It had been reported that HNF1A-AS1 (HNF1A Antisense RNA 1) was differently expressed in multiple tumors. The role of HNF1A-AS1 in colorectal cancer was less analyzed, and the mechanism of regulating the cell cycle has not been completely elucidated. Methods: Differentially expressed lncRNAs were screened out from the TCGA database. HNF1A-AS1 was examined in CRC clinical samples and cell lines by RT-qPCR. CCK8 assay, colony formation assay, flow cytometry, transwell assays, tube forming assay and vivo experiments were performed to study the function of HNF1A-AS1 in CRC tumor progression. Bioinformatic analysis, luciferase report assay, RNA pull-down and RIP assays were carried out to explore proteins binding HNF1A-AS1 and the potential downstream targets. Results: Our results showed that HNF1A-AS1 was upregulated in CRC and associated with unfavorable prognosis. HNF1A-AS1 promoted proliferation, migration and angiogenesis, accelerated cell cycle and reduced cell apoptosis in CRC. Bioinformatics prediction and further experiments proved that HNF1A-AS1 could promote CCND1 expression by suppressing PDCD4 or competitively sponging miR-93-5p. Meanwhile, METTL3 mediated HNF1A-AS1 m^6^A modification and affected its RNA stability. HNF1A-AS1/IGF2BP2/CCND1 may act as a complex to regulate the stability of CCND1. Conclusion: In summary, our result reveals the novel mechanism in which m^6^A-mediated HNF1A-AS1/IGF2BP2/CCND1 axis promotes CRC cell cycle progression, along with competitively sponging miR-93-5p to upregulate CCND1, demonstrating its significant role in cell cycle regulation and suggesting that HNF1A-AS1 may act as a potential prognostic marker of colorectal cancer in the future.

## 1. Materials and Methods

### 1.1. Bioinformatics of Gene Expression Database

RNA-sequencing datasets of CRC patients were downloaded from the TCGA website. Our inclusion criteria were as follows: (1) histopathologically confirmed colorectal cancer; (2) case-related clinical information, including age, gender, treatment, staging, survival time, etc.; (3) there was no distant metastasis before surgery; (4) other malignant tumors were excluded. A total of 52 paired colorectal samples were included, including 23 colorectal cancer samples in stage III/IV and 52 colorectal normal tissue samples. The whole process was performed based on the publication guidelines from the TCGA database.

### 1.2. Colorectal Clinical Tissues and CRC Cell Lines 

In total, 52 pairs of tissue specimens from CRC patients were collected in the study, including colorectal tissues and their respective adjacent mucosal tissues. The patients included in the study had accepted radical surgery in the Second Affiliated Hospital of Nanjing Medical University from 2012 to 2021. Pathological diagnosis and staging were evaluated based on the eighth edition cancer staging manual. Approved by the Ethics Committee of Nanjing Medical University (ethical approval number: KY No. 121), all the patients included agreed to join in the study and signed the relative informed consent.

Six colorectal cancer cell lines (HCT116, HT-29, SW480, SW620, DLD-1 and LOVO), human umbilical vein endothelial cells (HUVEC) and normal human colonic mucosal cell line (HcoEpic) were all from the Sciences cell bank of Chinese Academy. All these cell lines were cultivated in DMEM or RPMI1640, which were supplied with 10% fetal bovine serum (Gemini, Woodland, CA, USA), 100 mg/mL streptomycin and 100 U/mL penicillin.

### 1.3. Plasmid Construction and Cell Transfection

Three shRNAs targeting HNF1A-AS1, negative control, HNF1A-AS1 over-expressing plasmid, miRNA mimics and other targeted shRNAs were designed and purchased from GenePharma Company (Shanghai, China). They were transfected into CRC cells with lipo2000 (Invitrogen, Carlsbad, CA, USA) following the manufacturer’s manual. The sequences of all shRNAs were summarized in Table S1.

### 1.4. RNA Extraction and RT-qPCR

Total RNA from tissues and cells was extracted by the Trizol reagent (Invitrogen, USA) and reversely transcribed into cDNA, which acted as the template to run RT-qPCR through SYBR Green Master (Vazyme, Nanjing, China) according to the instructions from the manufacturer. The expression of relative genes was analyzed by the comparative threshold cycle (2−ΔΔCt) method and normalized to GAPDH. All the primers used were outlined in Table S2.

### 1.5. Immunoblotting 

Protein from the treated and untreated HCT116 and LOVO cells was extracted by RIPA (Beyotime, Nantong, China). The protein lysates were separated by SDS-PAGE and then transferred onto PVDF membranes. ECL chromogenic substrate was used for densitometry quantification after incubating specific antibodies at 4 °C for 12 h. Protein detection was performed using rabbit monoclonal anti-CCND1 (Abcam, Cambridge, UK, ab134175), anti-CDK4 (Abcam, ab108357), anti-P21 (Abcam, ab109520), anti-casepase3 (CST, #14220), anti-PARP (Proteintech, Rosemont, IL, USA, 13371-1-AP), anti-PDCD4 (Abcam, ab80590), anti-Vimentin (CST, #5741), anti-N-cadherin (Abcam, ab76011), anti-E-cadherin (Abcam, 76011), anti-METTL3 (Proteintech, 15073-1-AP), anti-IGF2BP2 (Proteintech, 11601-1-AP),anti-FLAG (Proteintech, 20543-1-AP), anti-pAKT (CST, #4060), anti-AKT (CST, #9272), anti-PI3K (CST, #4249), anti-pPI3K (CST, #4228). GAPDH (Affinity, West Bridgeford, UK, AF7021) was applied as the internal control.

### 1.6. Cell Proliferation and Transwell Assays

The CCK8 assay was used to determine the proliferation ability. A total of 2000–4000 transfected tumor cells were seeded into 96-well plates, and the CCK8 solution was added every 24 h to detect absorbance at 450 nm with a microplate reader. In colony formation, 500–1000 cells were seeded in 6-well plates and fixed with methanol and stained with 0.1% crystal violet after 14 days. A total of 5 × 10^4^ transfected cells were added to the upper chamber of insert (Millipore, Sandiego, CA, USA) and then fixed after 24–48 h. The number of formed colonies and cells migrating through the membrane were pictured and analyzed.

### 1.7. Cell Apoptosis and Cell Cycle Assays

The treated cells were collected after 48 h of transfection and resuspended with 100 uL binding buffer, mixed with 5 uL fluorescently labeled AnnexinV-FITC and 5 uL PI Staining Solution (Vazyme, China). The solution was incubated for 5–15 min and tested on the machine immediately after adding the binding buffer. As for cell cycle analysis, 75% ethanol was used to resuspend and fix cells overnight at −20 °C. Cells were stained by propidium iodide stain and incubated in darkness for 15 min, detected by flow cytometry (FACSCalibur, Becton Dickinson, Franklin Lakes, NJ, USA).

### 1.8. Tube Formation Assay

Briefly, 50 μL Matrigel (ABW-Bio, Shanghai, China) was seeded into 96-well plates (Nest, Wuxi, China) and put in a cell incubator for 30 min until Matrigel was coagulated. Then, 4 × 10^4^ vascular endothelial cells in 100 μL ECM (endothelial cell medium) (5% fetal bovine serum, 1% streptomycin and 1% ECGS) were added above Matrigel. Then, 100 μL supernatant of sh-NC cell and sh-HNF1A-AS1 cells was added into the plates, respectively. Within 12 h, the vascular endothelial cells stimulated by the supernatant grew and were pictured by the microscope. The length of the cell tubes was analyzed by ImageJ.

### 1.9. Dual Luciferase Reporter Assay

The related wild-type (WT) and mutant plasmids were constructed by Genebay company in Nanjing. The cells were plated in a 24-well plate. After 48 h of plasmid transfection, 200 uL lysis solution was added to each well at room temperature and incubated for 10 min. Next, the supernatant was collected after centrifugation, then100 uL of the supernatant was mixed with 100 uL of the luciferase reporter working solution.The firefly luciferase F value was tested on the machine and the renilla luciferase R value was measured after adding the terminating solution. The ratio of F/R was calculated for further analysis.

### 1.10. Tumor Xenograft Model 

The animal experiments were approved by the Institutional Animal Care and Use Committee at Nanjing Medical University. Ten BALB/C nude mice (4 weeks old, female) were injected in 1 × 10^6^ HCT116 cells in 100 uL PBS at each side. The tumor size was detected every four days after the injection of cells and calculated according to the formula (0.5 × tumor length × tumor short length^2^). Finally, the mice were scarified, and all tumors were stored for subsequent RNA extraction and IHC analysis.

### 1.11. RNA Immunoprecipitation (RIP) Assay

At least 2 × 10^7^ cells were collected and then lysed on ice in RIP lysis buffer according to the instructions provided by the Magna RIP Kit (Millipore, Sandiego, CA, USA). The lysates were conjugated with 5 ug of rabbit anti-IGF2BP2 (Proteintech, China) or 5 ug rabbit IgG antibody (Millipore, Sandiego, CA, USA) with magnetic beads in 1 mL wash buffer at 4 °C overnight. Then, the RNA-protein complex was digested and purified at 55 °C for half an hour after adding the proteinase K buffer. Total RNA was extracted by the Trizol reagent, and the expressions of related genes were detected by RT-qPCR. 

### 1.12. RNA Pull-Down Assay

The full-length plasmid of HNF1A-S1 was transcribed in vivo with mMESSAGE mMACHINE Kit (Thermo, Waltham, MA, USA), and then, the transcribed RNA was purified by MEGAclear Kit (Thermo, USA). Next, the RNA was labeled with biotin by 5′- and 3′-RACE Kit (Thermo, Waltham, MA, USA) based on the manufacturer’s instructions. A total of 50 pmol biotinylated RNA was incubated with A/G magnetic beads at room temperature for 30 min approximately. Then, about 6 × 10^6^ cells were lysed in 400 uL of IP lysis (Beyotime, Nantong, China) added with PMSF to ensure a sufficiently high protein concentration. The washed magnetic beads were incubated with protein lysate in 100 uL protein-RNA binding buffer (Thermo, Waltham, MA, USA) at 4 °C for 2 h. Finally, the proteins binding to RNA were washed and collected for Western blot and silver staining.

### 1.13. MeRIP qRT-PCR 

MeRIP was performed based on the previous protocol using the MeRIP m^6^A Kit (MerckMillipore, Darmstadt, Germany) [1,2]. Briefly, the total RNA was extracted by the Trizol regent and then treated with DNase I to avoid DNA contamination. A total of 100 ug purified RNA was fragmented into 300 bps by fragment reagents. After incubating A/G magnetic beads with anti-m^6^A antibody (Millipore, Sandiego, CA, USA) and anti-IgG for 30 min at room temperature, the fragmented RNA was added into the magnetic beads at 4 °C overnight in 500 uL of the RIP buffer. An amount of 10 ug RNA was saved as input. Then, the following procedure was the same as the RIP method.

### 1.14. RNA Stability Assay

An amount of 5 ug/mL Actinomycin D (Abmole, Houston, TX, USA) was added into the cells after 48 h of transfection; then, the cells were harvested at 0, 2, 4, 6, 8 h or 0, 20, 40, 60 and 80 min, respectively. Total RNA was extracted by Trizol, and the expression of related genes was analyzed by RT-qPCR normalized to GAPDH. The half-life of mRNA was calculated according to the related formula.

### 1.15. Fish 

The probe targeting HNF1A-S1 was designed by Ribo company (Guangzhou, China). Cells were fixed by 4% paraformaldehyde, and thencells were incubated with the probe at 37 °C overnight after permeabilization by PBS containing 0.5% Triton X-100 following the manufacturer’s instructions. The next day, cells were washed with SSC (Saline Sodium Citrate) buffer at 42 °C several times and incubated with anti-IGF2BP2 (Proteintech, 11601-1-AP, 1:200) overnight at 4 °C after blocking 30 minutes at room temperature. On the third day, the cells were washed and incubated with Alexa Fluor 488 anti-rabbit IgG (Abcam, 1:200, ab150077, Cambridge, UK). Nuclei were stained by DAPI, and then, the cells were pictured by confocal microscopy (LSM710, Jena, Thuringia, Germany).

### 1.16. Statistical Analysis

GraphPad Prism version 8.0 (Sandiego, CA, USA), Rstudio version 4.0.3 and Image J version 5.4 were used for data analysis and graphing. All experiments were independently repeated three times. The data differences between the two groups were analyzed by Student’s *t*-test, while ANOVA was used to compare more than two groups. The Chi-Square test was used to analyze clinicopathological data, and *p* < 0.05 was considered to be a significant difference.

## 2. Background

Malignant tumors have become the main origin of disease-related deaths around the world. Based on the latest statistics from the International Agency for Research on Cancer (IRIC), there were around 1,898,160 newly diagnosed cancer patients in the United States in 2021 [3]. The morbidity and mortality of colorectal cancer, a common gastrointestinal tumor, currently ranks third in both men and women [4]. Due to the insidious symptoms at initial onset, about 15% of the newly diagnosed CRC patients are accompanied by liver metastasis, and most of them lose the opportunity for radical surgery. The average 5-year survival rate of CRC is around 60%, and it had not been significantly improved with the widespread use of targeted drugs and immunotherapy. Thus, it is urgent to explore new markers that can effectively screen tumors, guide treatment and predict prognosis. Long non-coding RNAs are the special kind of RNAs with more than 200 nucleotides in length, which have no ability to encode proteins. They could modulate gene expression through chromatin modification, transcriptional regulation, post-transcription modification and in other ways [5]. 

LncRNAs play biological roles partly through competitively sponging microRNAs. The complementary sequences between microRNAs and target RNA transcripts, which is known as RISC (programed RNA-induced silencing complex), could silence the gene expression of target genes [6]. The competitive binding relationship also exists between lncRNAs and miRNAs. LncRNA PVT1 regulates the downstream target gene RUNX2 not only through the PVT1/miR-30d-5p/RUNX2 axis [7] but also through the PVT1/miR-455/RUNX2 axis [8,9]. As the most common epigenetic modification in eukaryotic cells, N6-methylation occurs in nearly 90% of RNAs, and it not only regulates mRNA transcription, processing, degradation and translation but also modulates much non-coding RNAs (ncRNAs). m^6^A modification takes part in the progression of multiple diseases, such as heart failure, glioblastoma, colorectal cancer and so on [2,10,11,12,13]. As a dynamic and reversible process, m^6^A methylation modification is mainly mediated by methyltransferases, demethylases and methylation recognition proteins, whose functions are to increase, remove and recognize the m^6^A methylation spot, respectively. There are increasing research works regarding m^6^A methylation modulation in lncRNAs. LncRNA FAM225A was upregulated in nasopharyngeal carcinoma (NPC) tumor tissue, and high expression of FAM225A was related with poor clinical prognosis. MeRIP experiments revealed that after silencing METTL3, the m^6^A level of FAM225A decreased, along with the decreased stability and expression of FAM225A [14]. There have been studies about the pivotal role of HNF1A-AS1 in NSCLC, hepatocellular carcinoma and gastric cancer [15,16,17,18,19]. In CRC, studies have shown that HNF1A-AS1 mainly played biological roles through competitively interacting with microRNAs, such as miR-34a, miR-124 and so on [20,21]. 

Our research explored the mechanisms of HNF1A-AS1 regulating the cell cycle in CRC deeply, which had not been studied previously. Functional assays revealed that HNF1A-AS1 could promote proliferation, migration and angiogenesis in CRC. Mechanically, we found that HNF1A-AS1 could sponge miR-93-5p by forming the HNF1A-AS1/miR-93-5p/AGO2 complex to upregulate CCND1, and it could stabilize the CCND1 mRNA mediated by METTL3-induced m^6^A modification as well. HNF1A-AS1 could also suppress PDCD4 to ultimately regulate CCND1 by activating the PI3K/AKT pathway. Our results revealed that HNF1A-AS1 had the potential to be a diagnosis target and novel prognosis biomarker in colorectal cancer.

## 3. Results

### 3.1. HNF1A-AS1 Was Upregulated in Human CRC Tissues and Correlated with Poor Prognosis 

**Figure 1:**
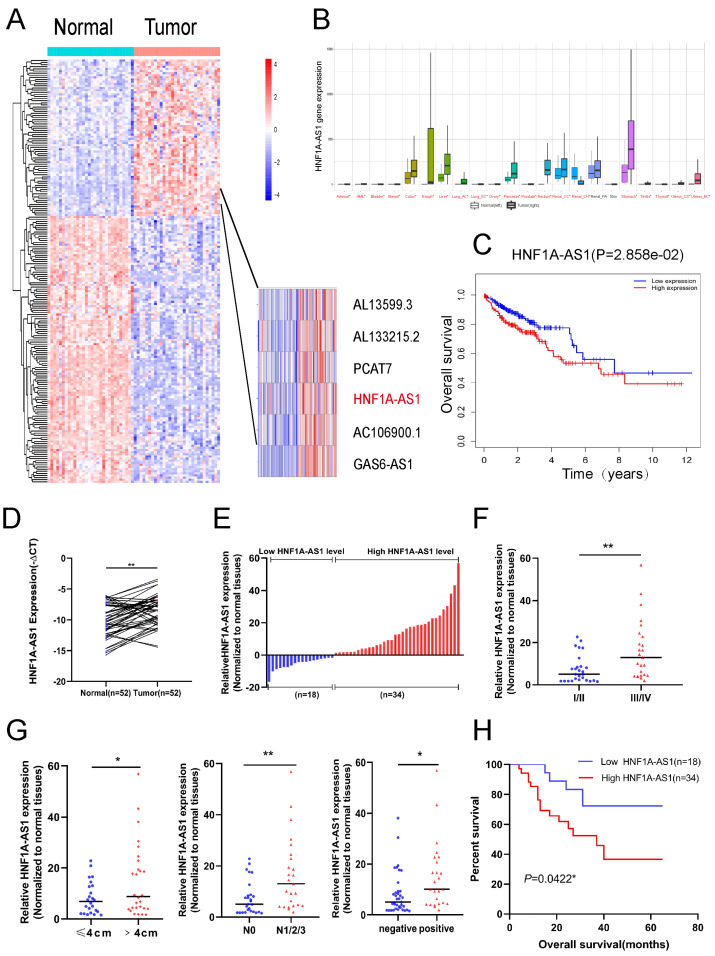
Expression of HNF1A-AS1 in colorectal cancer and its clinical characteristics. (**A**) Heatmap of differentially expressed lncRNAs in CRC patients with stage (III/IV) compared with normal tissues from TCGA-CRC database. (**B**) The overall expression of HNF1A-AS1 in multiple human cancers from TCGA. (**C**) Kaplan–Meier survival curves for HNF1A-AS1 in CRC patients from TCGA database. (**D**) HNF1A-AS1 was highly expressed in CRC tissues (*n* = 52) compared with normal tissues through RT-qPCR detection (−ΔCT). (**E**) Detailed expression of HNF1A-AS1 was detected in 52 pairs of CRC tissues and classified into relatively high-expression and low-expression group through real-time qPCR. (**F**) Patients with higher pathological stage (III/IV) had higher level of HNF1A-AS1 expression than patients with lower pathological stage (I/II). (**G**) HNF1A-AS1 was higher expressed in patients with large tumor (>4 cm), lymph node metastasis (N1/2/3) or distant metastasis. (**H**) Kaplan–Meier analysis of the survival curves for CRC patients in low HNF1A-AS1 (*n* = 18) expression and high HNF1A-AS1 (*n* = 34) expression. ** *p* < 0.01 * *p* < 0.05.

To explore the potential long non-coding RNAs influencing colorectal cancer progression, we screened out 30 tumor patients with stage III/IV and 30 corresponding normal patients totally from the TCGA-CRC database. After differential analysis by edgeR, we picked up 200 potential long non-coding RNAs for further exploration based on their base expression of genes and *p* values. Finally, six highly expressed lncRNAs were chosen for further exploration (Figure 1A). The expressions of PCAT7 and GAS6-AS1 were shown lower than HNF1A-AS1 in several human CRC cell lines (Figure S1A). According to the TCGA database, HNF1A-AS1 was highly expressed in several cancers, including colon cancer and rectum cancer, suggesting its role as an oncogene (Figure 1B). Additionally, high expression of HNF1A-AS1 was related with poor overall survival based on the TCGA database (Figure 1C). In our 52 paired CRC patients’ samples, 70% of whom did not have positive metastasis and most of whom were diagnosed at an early stage, HNF1A-AS1 was higher expressed in tumor tissues than in normal tissues based on RT-qPCR results (Figure 1D). HNF1A-AS1 expression was upregulated in 65.4% (34 out of 52) of CRC tissues, as shown in Figure 1E. Subsequently, we then analyzed the relationship between HNF1A-AS1 and clinicopathologic factors in our samples (Table 1). The results demonstrated that high expression of HNF1A-AS1 was correlated with higher pathological stage (III/IV), positive lymph node metastasis and distant metastasis (Figure 1F,G). Meanwhile, patients with a high expression of HNF1A-AS1 showed shorter overall survival time than low-expression patients through the Kaplan–Meier analysis (Figure 1H). In conclusion, the high expression of HNF1A-AS1 was correlated with unfavorable prognosis in CRC patients.

### 3.2. HNF1A-AS1 Promotes Progression of CRC In Vitro 

**Figure 2:**
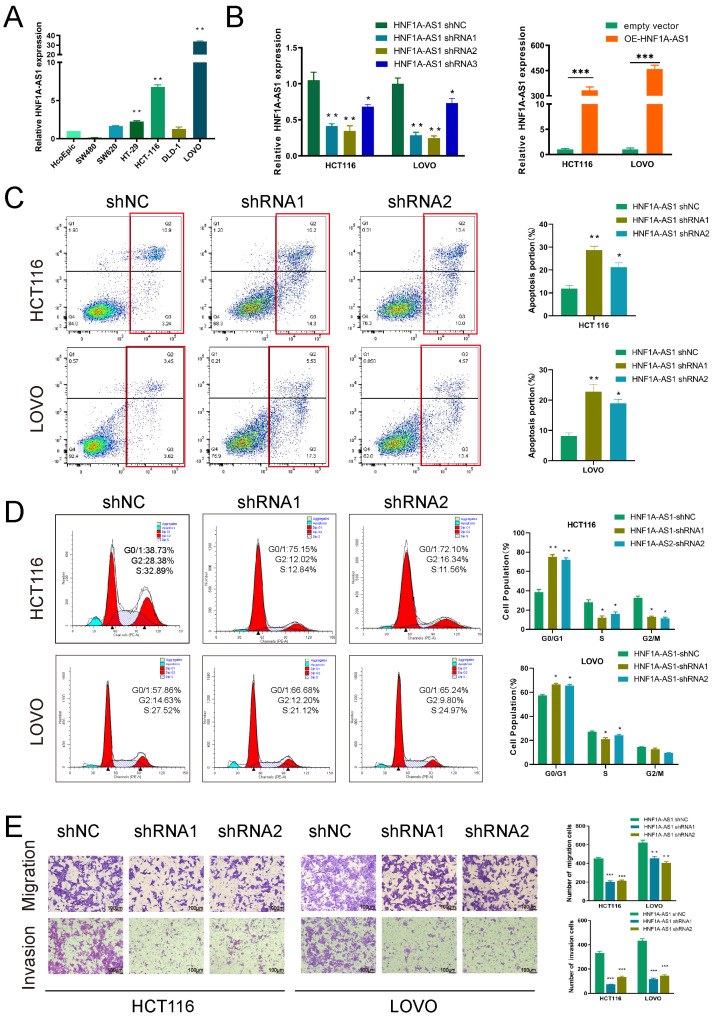
HNF1A-AS1 promoted CRC cell proliferation in vitro. (**A**) Expression of HNF1A-AS1 in several CRC cell lines and normal human colon epithelial cell was detected through RT-qPCR. (**B**) The knockdown and over-expressing efficiency of HNF1A-AS1 were detected by RT-qPCR. (**C**) Flow cytometry assays were performed to observe the change of percentage of apoptosis cells after silencing HNF1A-AS1. (**D**) Flow cytometry was carried out to compare the differences in cell cycle progression when CRC cells were transfected with shNC and shHNF1A-AS1. The bar graph represents percentage data for G0/G1, S and G2/M phase cells. (**E**) Transwell assays were performed to determine the effects of HNF1A-AS1 on cell migration and invasion in shHNF1A-AS1 or shNC-transfected HCT116 and LOVO cells. Scare bar:100μm * *p* < 0.05, ** *p* < 0.01, *** *p* < 0.001.

**Figure 3:**
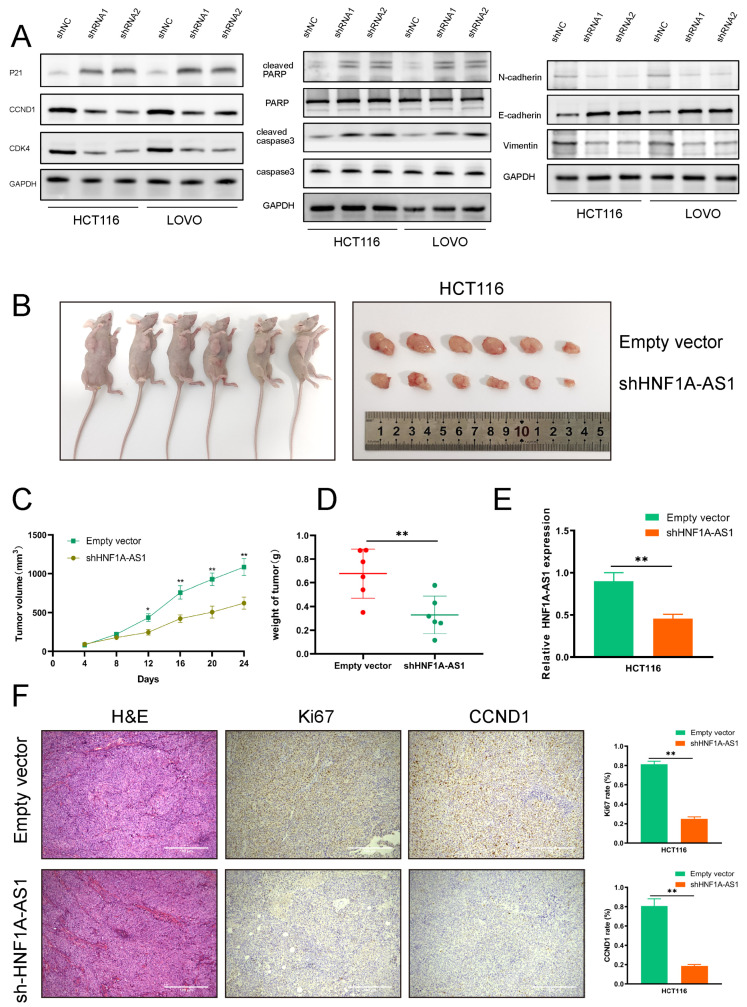
HNF1A-AS1 accelerated tumor growth of CRC in vivo. (**A**) Cell cycle check point proteins (p21, CCND1, CDK4), apoptosis-related proteins (caspase3, cleaved-caspase3, PARP, cleaved PARP) and epithelial-mesenchymal transition (EMT)-associated proteins (N-cadherin, E-cadherin, Vimentin) were detected in HCT116 and LOVO cells transfected with shHNF1A-AS1. (**B**) Nude mice were injected with HCT116 cells transfected with empty vectors and sh-HNF1A-AS1. (**C**) Tumor volumes were measured and recorded every 4 days after injection. (**D**) Tumors were removed after 24 days, and their weights had significant differences. (**E**) The expression of HNF1A-AS1 from empty vector group and shHNF1A-AS1 group was detected through real-time qPCR. **(F)** Tumors from shHNF1A-AS1 group had lower Ki67 and CCND1 expression than empty vector group. Scar bar: 100 μm, * *p* < 0.05, ** *p* < 0.01.

Firstly, we detected the expression of HNF1A-AS1 in representative colorectal cancer cell lines by RT-qPCR, and the result suggested that it was upregulated in HT-29, HCT116 and LOVO cell lines compared with the human normal colon epithelial cell (HcoEpic) (Figure 2A). Thus, we selected HCT116 and LOVO cell lines for further research. Then, we designed the HNF1A-AS1 expression vector and three shRNAs (small hairpin RNAs) to explore its biological role in CRC in which shRNA1 and shRNA2 had a better knockdown efficiency and were chosen for later experiments (Figure 2B). The proliferation ability of HNF1A-AS1 was examined through CCK8 and colony formation experiments (Figure S1B,C). It was shown that a knockdown of HNF1A-AS1 significantly suppressed cell viability, while over-expression of HNF1A-AS1 increased cell viability. Meanwhile, the fraction of apoptotic cells was increased significantly (Figure 2C), and cell cycle was suppressed at the G0/G1 phase after silencing HNF1A-AS1 in HCT116 and LOVO cells through flow cytometry (Figure 2D). Our results also demonstrated that a knockdown of HNF1A-AS1 inhibited the cell migration and invasion abilities in both cell lines (Figure 2E), while over-expression of HNF1A-AS1 augmented the migration and invasion ability (Figure S1D). Tube formation assays were performed to explore cell angiogenesis ability. Results revealed that silencing HNF1A-AS1 weakened angiogenesis, while over-expressing HNF1A-AS1 promoted angiogenesis (Figure S1E). Next, we detected the relevant proteins regulating cell cycle (p21, G1-S check point protein like CCND1 and CDK4), cell death (caspase3, PARP) and EMT, which further supported our results (Figure 3A). CCND1 and CDK4 (cyclin-dependent kinase 4), which promoted the G1-S phase point transmission, were downregulated when silencing HNF1A-AS1, indicating that a knockdown of HNF1A-AS1 restrained the cell cycle progression. As a typical tumor suppression gene, p21 was upregulated after a knockdown of HNF1A-S1, supporting the role of HNF1A-AS1 in promoting the cell cycle further. Consistent with the apoptosis results, sh-HNF1A-AS1 cells expressed a significantly higher level of apoptosis-related proteins, including cleaved PARP and cleaved caspase3. In addition, N-cadherin and Vimentin proteins, playing important roles in EMT (Epithelial-mesenchymal transition), were decreased, while E-cadherin was higher expressed in sh-HNF1A-AS1-treated CRC cells. These investigations suggested that HNF1A-AS1 exerted critical influences on CRC cells by affecting the cell cycle, apoptosis and migration. Overall, our results demonstrated that HNF1A-AS1 promoted CRC cell progression in vitro.

### 3.3. HNF1A-AS1 Promotes CRC Progression In Vivo 

HCT116 cells were transfected with empty vector or shHNF1A-AS1 to establish the tumor model in four-week-old BALB/c nude mice to explore HNF1A-AS1’s biological role in vivo. The treated cells were injected into both axilla of the same nude mouse, respectively (Figure 3B). The volumes of the tumors were measured every 4 days. Twenty-four days after injection, the shHNF1A-AS1 group showed a lower speed of tumor growth contrasted with the empty vector group (Figure 3C), as well as lower tumor volume and weight (Figure 3D). The expression of HNF1A-AS1 was reduced in the shHNF1A-AS1 group compared with the empty vector group based on RT-qPCR results (Figure 3E). Furthermore, IHC analysis revealed that tumors from the HNF1A-AS1-knockdown group had a weaker expression of Ki67 and CCND1 than those in the empty vector group (Figure 3F). Therefore, our results revealed that HNF1A-AS1 could promote CRC progression in vivo.

### 3.4. HNF1A-AS1 Affects CRC Progression through Regulating the Expression of CCND1 and PDCD4 

**Figure 4:**
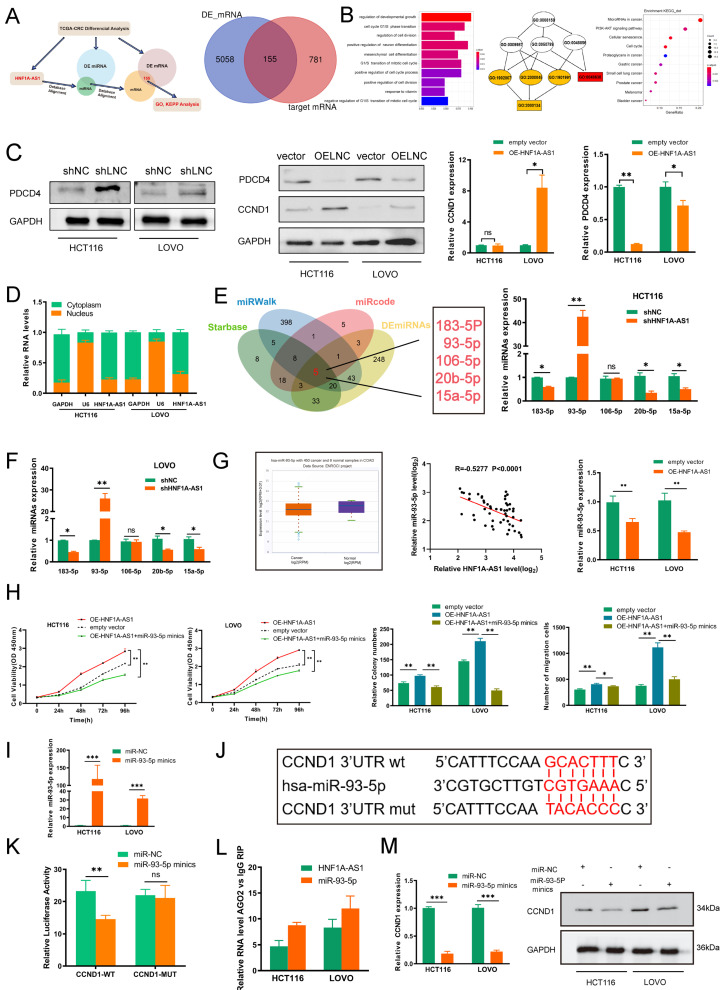
HNF1A-AS1 suppressed PDCD4 and sponged miR-93-5p to upregulate CCND1. (**A**) In total, 155 predicted downstream mRNAs were obtained by bioinformatics analysis. (**B**) The biological function of predicted downstream genes was classified through GO and KEGG analysis. (**C**) CCND1 and PDCD4 were screened as the targeted genes and confirmed through RT-qPCR and Western blot in shHNF1A-AS1 and over-expressing HNF1A-AS1 CRC cells. (**D**) The distribution of HNF1A-AS1 in the cytoplasm and nucleus was detected through RT-qPCR in HCT116 and LOVO cells. U6 acted as the nuclear control, while GAPDH acted as the cytoplasm control. (**E**) Potential microRNAs binding HNF1A-AS1 with CCND1 were predicted though Starbase, miRWalk, miRcode websites and differently expressed miRNAs from TCGA. Five microRNAs were overlapped and examined in the shHNF1A-AS1 HCT116 cell line. (**F**) Five microRNAs (miR-183-5p, miR-93-5p, miR-106-5p, miR-20b-5p, miR-15a-5p) were detected in another shHNF1A-AS1 CRC cell line (LOVO). (**G**) The expression of miR-93-5p in CRC cancer and normal samples from TCGA database. The negative relation between HNF1A-AS1 and miR-93-5p was detected through RT-qPCR in tumor tissues and over-expressing HNF1A-AS1 cell lines. (**H**) Over-expressing miR-93-5p abolished the proliferation and migration induced by over-expressing HNF1A-AS1 through CCK8 and transwell assays. (**I**) The over-expressing efficiency of miR-93-5p was detected through RT-qPCR in HCT116 and LOVO cells. (**J**) Construction of wild-type and mutant type CCND1 plasmids based on the predicted binding sites from Starbase website. (**K**) Dual luciferase activity in HEK-293 T cells co-transfected with the wild-type or mutant-type CCND1 plasmid and miR-93-5p mimics. (**L**) HNF1A-AS1 and miR-93-5p were bound to AGO2 through RIP experiment. (**M**) The relation between miR-93-5p and CCND1 was analyzed by RT-qPCR and Western blot. * *p* <0.05, ** *p* < 0.01, *** *p* < 0.001.

Apart from the differently expressed long non-coding RNAs, after analyzing 30 CRC patients in stage III/IV and 30 normal patients, 356 differentially expressed miRNAs (DEmiRNAs) and 5213 differentially expressed mRNAs (DEmRNAs) were also obtained from the same TCGA-CRC database. A total of 44 microRNA families aligned with HNF1A-AS1 were predicted from the miRcode website; 14 miRNAs were obtained by intersecting the matched miRNAs with 356 DEmiRNAs. Then, we obtained 936 mRNAs through predicting the aligned mRNA of these 14 miRNAs in miRDB, TargetScan and other websites. Intersecting these 936 mRNAs with 5213 differentially expressed mRNAs, we obtained 155 mRNAs, which could be the potential downstream genes of HNF1A-AS1 (Figure 4A). After the GO cluster analysis of the 155 mRNAs, it was found that the top three items clustered were growth and development regulation, cell cycle G1/S phase transition and regulation of cell division. In the KEGG pathway clustering analysis, the top three entries were tumor microRNAs, PI3K-Akt signaling pathway and cellular senescence (Figure 4B). 

The clustered genes among the top three entries (CCND1, PDCD4, VEGFA, SOX4 and so on) in the biological process were examined by RT-qPCR after silencing HNF1A-AS1. The results showed the expression of CyclinD1 (CCND1) was downregulated, and programed cell death 4 (PDCD4) was increased in both cell lines (Figure S2A). According to the analysis from the TCGA database, CCND1 was highly expressed, while PDCD4, as a tumor suppressor gene, was less expressed in COAD and READ than normal tissues (Figure S2B). Additionally, CCND1 and PDCD4 were both significant genes regulating the cell cycle, which corresponded to the KEGG analysis. We then further explored the protein expression of CCND1 and PDCD4 through Western blot assays. Our results suggested that CCND1 was downregulated (Figure 3A), and PDCD4 was upregulated after knocking down HNF1A-AS1 (Figure 4C). Meanwhile, the expression of CCND1 increased, and PDCD4 decreased after over-expressing HNF1A-AS1. The mRNA expression of CCND1 and PDCD4 had similar change in the cell lines (Figure 4C). Thus, we chose CCND1 and PDCD4 as the downstream target genes of HNF1A-AS1 for further exploration. We performed rescue experiments to further clarify the regulatory roles of CCND1 and PDCD4 in HNF1A-AS1. The relative protein expression of CCND1 and PDCD4 in the rescue assays was shown in Figure S2C. It turned out that the proliferation ability of CRC cells was restored after the over-expression of CCND1 or knockdown PDCD4 in CRC cells induced by silencing HNF1A-AS1 through CCK8 and colony formation assays. Similarly, over-expression of CCND1 or silencing PDCD4 could reverse the migration ability caused by silencing HNF1A-AS1 (Figure S2D,E). From the results above, we confirmed that HNF1A-AS1 could regulate CRC progression through regulating CCND1 and PDCD4.

### 3.5. HNF1A-AS1 Sponges miR-93-5p to Upregulate CCND1

We performed subcellular fraction experiments to identify the location of HNF1A-AS1 in HCT116 and LOVO cell lines to explore its specific mechanism (Figure 4D). HNF1A-AS1 was distributed in both the nucleus and the cytoplasm, while its ratio in the cytoplasm was much higher. The result implied that HNF1A-AS1 could participate in the transcriptional and post-transcriptional regulation of downstream genes. It was verified that lncRNAs could regulate downstream gene expression by competitively sponging specific microRNAs in the cytoplasm. Therefore, we used the Starbase and miRWalk websites to predict the potential miRNAs binding CCND1 while using miRcode to predict the potential miRNAs binding HNF1A-AS1. After intersecting these results with 356 differentially expressed miRNAs, we screened out 5 miRNAs, which could interact with HNF1A-AS1 and CCND1 in the meantime (Figure 4E). Then, we detected the expression of five miRNAs after silencing HNF1A-AS1 in HCT116 and LOVO cell lines. The result showed that only the expression of miR-93-5p increased, while the others decreased or had no change (Figure 4E,F). Therefore, we chose miR-93-5p for furtherstudy. miR-93-5p was highly expressed in normal tissues from the TCGA database, and a correlation analysis of 52 CRC samples using RT-qPCR showed that HNF1A-AS1 was negatively correlated with miR-93-5p. At the same time, we also verified that the expression of miR-93-5p decreased when over-expressing HNF1A-AS1 (Figure 4G). Then, we carried out the functional recovery experiments to explore the effect of miR-93-5p on HNF1A-AS1. miR-93-5p over-expression significantly suppressed the cell proliferation ability mediated by over-expressing HNF1A-AS1 through cck8 and colony formations. Similarly, the migration ability of CRC cells was weakened after over-expressing miR-93-5p (Figure 4H and Figure S2F).

The over-expression efficiency of miR-93-5p was examined by real-time qPCR (Figure 4I). We constructed wild-type and mutant plasmids of CCND1 based on the high-scoring binding site according to the Starbase prediction results (Figure 4J). The dual luciferase reporter experiment result in the HEK293T cell demonstrated that luciferase activity declined after co-transfection of miR-93-5p mimics and CCND1-3’UTR-WT, while it had no significant change after co-transfection of CCND1- 3’UTR-MUT and miR-93-5p mimics, suggesting the direct interaction between miR-93-5p and CCND1 (Figure 4K). miRNAs were confirmed to regulate RNA degradation or translational suppression through interacting with their target genes in a AGO2-dependent way. Therefore, we conducted the RIP assay to find that AGO2 could combine with HNF1A-AS1 and miR-93-5p, illustrating that HNF1A-AS1 could affect CCND1 through forming the HNF1A-AS1/AGO2/miR-93-5p complex (Figure 4L). Additionally, CCND1 was decreased in both the RNA and the protein level after over-expressing miR-93-5p in HCT116 and LOVO cell lines (Figure 4M). In summary, HNF1A-AS1 could promote CCND1 through sponging miR-93-5p in the cytoplasm.

### 3.6. HNF1A-AS1/IGF2BP2/CCND1 Complex Stabilizes CCND1 mRNA

**Figure 5:**
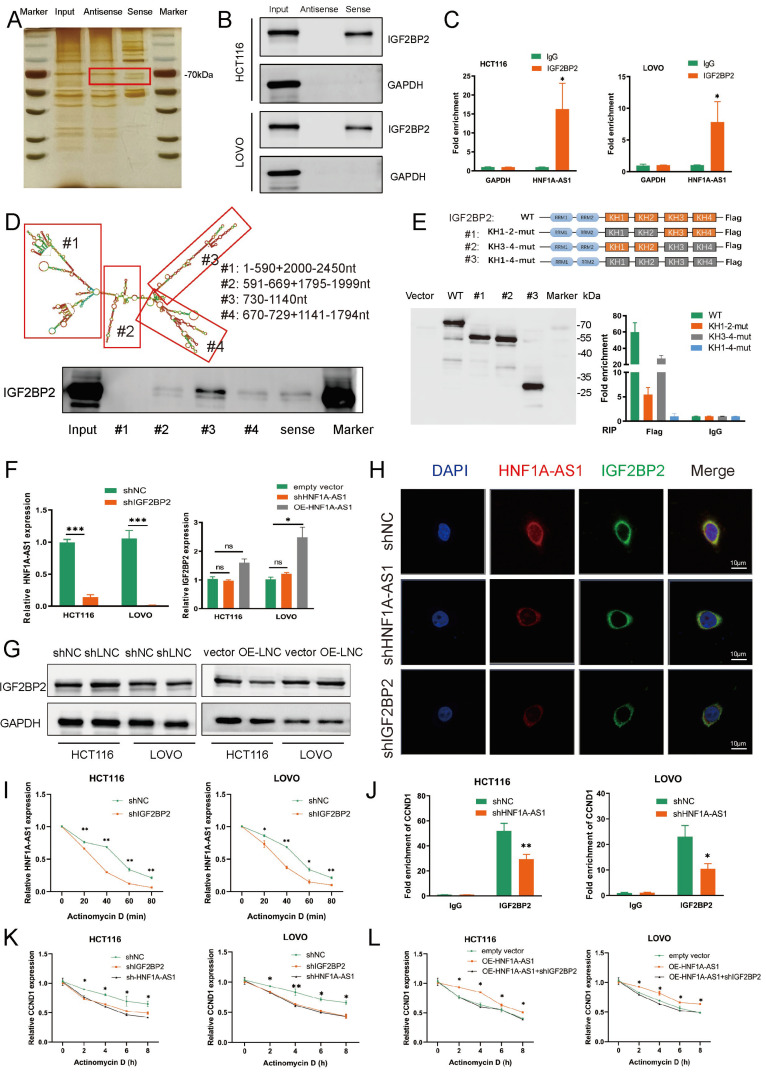
HNF1A-AS1/IGF2BP2/CCND1 complex stabilized CCND1 mRNA. (**A**) RNA pull-down assays were carried out using HNF1A-AS1 sense and antisense plasmids incubated with total protein extracts from CRC cells, followed by silver staining. The red box indicates differently expressed proteins. (**B**) Western blot to confirm the specific interaction between IGF2BP2 and biotinylated HNF1A-AS1, with GAPDH serving as negative control. (**C**) RIP was performed to examine the enrichment between HNF1A-AS1 and IGF2BP2 in HCT116 and LOVO cells. GAPDH served as the negative control. (**D**) Secondary structure of HNF1A-AS1 was predicted from RNAfold website. Several deletions of HNF1A-AS1 were designed to explore the core regions of HNF1A-AS1 required for the specific interaction with IGF2BP2 in the RNA pull-down assays. (**E**) Schematic structures show different IGF2BP2 variants used in our study, and the molecular weights of the designed mutants were confirmed through immunoblot of anti-FLAG. RIP analysis was then performed to find the binding domains in HEK293T cell transfected with FLAG-tagged full-length or mutated IGF2BP2. (**F**) RT-qPCR analysis of the HNF1A-AS1 expression change in CRC cell lines transfected with shNC or shIGF2BP2. IGF2BP2 mRNA level was also examined through RT-qPCR in CRC cell lines transfected with shHNF1A-AS1 or OE-HNF1A-AS1 (over-expressing HNF1A-AS1 plasmid). (**G**) IGF2BP2 expression in CRC cells transfected with shHNF1A-S1 or OE-HNF1A-AS1 was analyzed through Western blot assays. (**H**) FISH showed the colocalization of HNF1A-SA1 and IGF2BP2 in the shHNF1A-AS1 and shIGF2BP2 cells. Scale bars: 10μm (**I**) IGF2BP2 knockdown decreased the stability of HNF1A-AS1 through RNA stability assay after adding Actinomycin D at 0,20,40,60 and 80 min in CRC cell lines. (**J**) The enrichment of CCND1 with IGF2BP2 was downregulated after silencing HNF1A-AS1 through RIP analysis in CRC cell lines. (**K**) Negative control, HNF1A-AS1 knockdown and IGF2BP2 knockdown cells were treated with 5 mg/mL Actinomycin D at the corresponding time. Total RNA was extracted, and the mRNA half-life time of CCND1 was calculated by RT-qPCR normalized to GAPDH. (**L**) mRNA stability of CCND1 was detected in the vector, HNF1A-AS1-over-expressing with or without IGF2BP2-kockdown cells in CRC cell lines. * *p* < 0.05, ** *p* < 0.01, *** *p* < 0.001.

Apart from sponging miRNAs to play biological roles, lncRNAs could also interact with RNA binding proteins (RBPs) to regulate downstream genes. Then, we performed the RNA pull-down assays to explore the potential RBPs interacting with HNF1A-AS1. Silver staining showed that there was a significantly differently expressed protein around 70 kDa between sense-HNF1A-AS1 and antisense-HNF1A-AS1 (Figure 5A). Mass spectrometry confirmed the protein as IGF2BP2(67 kDa); thus, IGF2BP2 was selected for further study. The interaction between HNF1A-AS1 and IGF2BP2 was further confirmed through Western blot (Figure 5B). RIP (RNA immunoprecipitation) experiments also verified the direct interaction between HNF1A-AS1 and IGF2BP2 (Figure 5C). To investigate the binding site in HNF1A-AS1 in depth, we designed four deletion mutants according to the secondary structure of HNF1A-AS1 predicted from RNAfold. Our results revealed that #3(730–1140 nt) of the HNF1A-AS1 transcript interacted with IGF2BP2 much more strongly than other parts (Figure 5D). Next, we designed several IGF2BP2 mutants, mainly focusing on KH domains, to explore which domain of IGF2BP2 played the most important role between their interactions. The molecular weight of these mutants was detected through Western blot in the HEK293T cell. Further RIP assays demonstrated that the RRM domain did not interact with HNF1A-AS1, and the KH1-2 domain was much more indispensable than the KH3-4 domain for the interaction between HNF1A-AS1 and IGF2BP2 (Figure 5E). 

Interestingly, we found that HNF1A-AS1 failed to affect IGF2BP2 expression through RT-qPCR and Western blot, indicating that IGF2BP2 was not the target gene of HNF1A-AS1. On the other hand, HNF1A-AS1 decreased significantly when silencing IGF2BP2 (Figure 5F,G). Their relationship was further confirmed in FISH assays. HNF1A-AS1 did not affect the IGF2BP2 cellular localization, while a knockdown of IGF2BP2 weakened HNF1A-AS1 fluorescence intensity (Figure 5H). Additionally, IGF2BP2 decreased HNF1A-AS1 stability in HCT116 and LOVO cells (Figure 5I). We next explored the role of the HNF1A-AS1/IGF2BP2 axis in CRC and carried out rescue assays. Subsequently, IGF2BP2 knockdown abolished proliferation and migration in HCT116 and LOVO cells elicited by over-expressing HNF1A-AS1, suggesting that IGF2BP2 mediated HNF1A-AS1-induced proliferation and migration in CRC cells (Figure S3A,B).

IGF2BP2, as an m^6^A “reader” and conserved RNA binding protein (RBP), has been reported to stabilize a large number of target mRNAs. Therefore, we hypothesized that HNF1A-AS1 could cooperate with IGF2BP2 to stabilize CCND1 stability. According to the TCGA database, IGF2BP2 was higher expressed in CRC tumor tissues (Figure S3C). Then, we knocked down IGF2BP2 (Figure S3D) and found that the mRNA expression of CCND1 decreased based on RT-qPCR results (Figure S3E). Moreover, the over-expression of IGF2BP2 increased the downregulation of CCND1 induced by HNF1A-AS1 knockdown based on RT-qPCR and Western blot assays (Figure S3F). CCND1 mRNA could interact with IGF2BP2 as well in RIP assay results. The enrichment of CCND1 decreased when knocking down HNF1A-AS1, indicating that HNF1A-AS1 regulated CCND1 through combining with IGF2BP2 (Figure 5J). Then, we explored whether the HNF1A-AS1/IGF2BP2/CCND1 complex regulated CCND1 expression through stabilizing CCND1 mRNA stability. CCND1 mRNA stability decreased after silencing HNF1A-AS1 or IGF2BP2 (Figure 5K). IGF2BP2 knockdown attenuated the CCND1 mRNA stability mediated by over-expressing HNF1A-AS1 (Figure 5L). In conclusion, our result indicated that HNF1A-AS1 enhanced CCND1 mRNA stability by cooperating with IGF2BP2, ultimately promoting CCND1 expression.

### 3.7. METTL3 Mediates HNF1A-AS1 m^6^A Modification and Contributes to Its Upregulation in CRC

**Figure 6:**
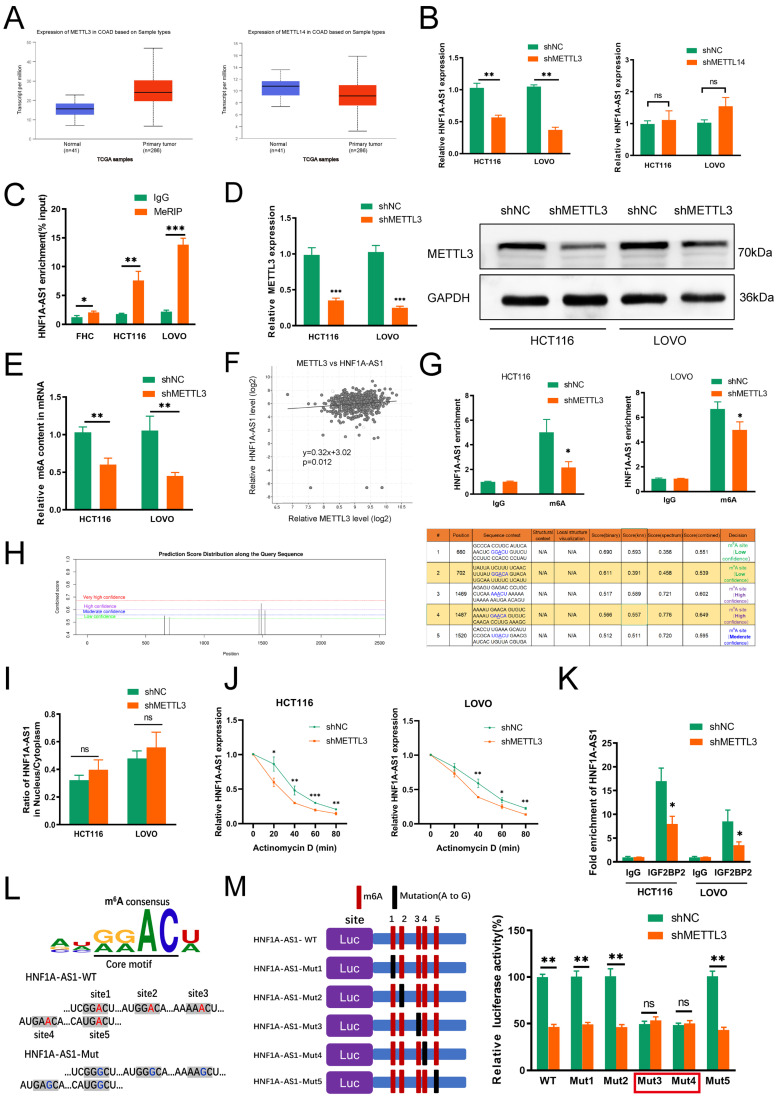
METTL3 induced HNF1A-AS1 m^6^A modification and upregulation in CRC. (**A**) The expression of METTL3 and METTL14 in colorectal cancer from TCGA database. (**B**) The expression of HNF1A-AS1 in CRC cells transfected with shMETTL3 or shMETTL14 through real-time qPCR analysis. (**C**) MeRIP-qPCR analysis of HNF1A-AS1 in FHC (normal human colon epithelial cell), HCT116 and LOVO cells. (**D**) Knockdown efficiency of METTL3 was confirmed through RT-qPCR and Western blot in HCT116 and LOVO cells. (**E**) Total m^6^A content in HCT116 and LOVO cells transfected with negative control or shMETTL3 was detected through colorimetric determination. (**F**) Correlation between HNF1A-AS1 and METTL3 from the TCGA-CRC database. (**G**) MeRIP-qPCR was performed to detect the change of m^6^A level in HNF1A-AS1 after silencing METTL3 in HCT116 and LOVO cells. (**H**) Potential m^6^A modified sites in HNF1A-AS1 predicted in SRAMP website. (**I**) The distribution of HNF1A-AS1 in negative control and METTL3-knockdown CRC cells through nuclear-cytoplasmic fraction experiments analysis, U6 and GAPDH acting as the controls in nucleus and cytoplasm, independently. (**J**) Negative control and METTL3-knockdown cells were treated with 5 mg/mL Actinomycin D every 20 min. Total RNA was extracted, and the mRNA half-life time of HNF1A-AS1 was calculated by RT-qPCR normalized to GAPDH. (**K**) The enrichment of HNF1A-AS1 on IGF2BP2 in CRC cells transfected with shMETTL3 through RIP analysis. (**L**,**M**) Mutated HNF1A-AS1 of pmirGLO vector was represented to explore the m^6^A roles in HNF1A-AS1 expression. The luciferase activities of different mutated HNF1A-AS1 plasmids were detected in indicated groups. * *p* < 0.05, ** *p* < 0.01, *** *p* < 0.001.

m^6^A modification played critical roles in numerous biological processes, such as RNA stability, location, protein translation and so on. METTL3 and METTL14, the major ingredients of the “writer” complex, played an irreplaceable function in methylation dynamic progression; thus, we analyzed the expression of METTL3 and METTL14 in CRC from the TCGA dataset. METTL3 was shown to be much more highly expressed in CRC tumor tissues, while METTL14 was much more expressed in normal CRC tissues (Figure 6A). Meanwhile, METTL3 and METTL14 were both reported to regulate CRC progression; thus, we detected the expression of HNF1A-AS1 by silencing METTL3 or METTL14. HNF1A-AS1 was downregulated after silencing METTL3. However, there was no significant change in HNF1A-AS1 expression after knocking down METTL14 (Figure 6B). The m^6^A enrichment of HNF1A-AS1 was higher in HCT116 and LOVO cell lines than in the human normal colon epithelial cell (Figure 6C), showing that m^6^A methylation occurred in the HNF1A-AS1 transcript. Then, we knocked down METTL3 (Figure 6D), and the total m^6^A content of the two cell lines was decreased (Figure 6E). Meanwhile, METTL3 had a positive relation with HNF1A-AS1 based on the TCGA analysis (Figure 6F).

Then, the MeRIP experiments were carried out, and the result revealed that the relative m^6^A methylation enrichment of HNF1A-AS1 was decreased after METTL3 knockdown (Figure 6G). There were five potential m^6^A modification sites in the HNF1A-AS1 sequence, two of which (position: 1469 and 1487) were assessed with high confidence predicted from the SRAMP website (Figure 6H). To explore the specific mechanism contributing to the m^6^A-mediated upregulation of HNF1A-AS1 in depth, we carried out the associated assays. The nucleus–cytoplasm fraction assay results suggested that the localization of HNF1A-AS1 in HCT116 and LOVO cell lines was not changed after knocking down METTL3 (Figure 6I). Then, we detected the influence of METTL3 knockdown on HNF1A-AS1 stability. The half-life of HNF1A-AS1 decreased significantly during 80 minutes after treatment with METTL3 shRNA (Figure 6J), indicating that METTL3 modulated HNF1A-AS1 expression by affecting HNF1A-AS1 stability. RIP analysis indicated that HNF1A-AS1 was enriched in the IGF2BP2 protein, and METTL3 knockdown decreased HNF1A-AS1 enrichment with IGF2BP2, suggesting that METTL3-induced m^6^A modification regulated the interaction of HNF1A-AS1 with IGF2BP2 (Figure 6K). To explore which binding sites played the main role in methylation, we designed five mutants based on the predicted binding sites (Figure 6L). Next, we performed the luciferase report assays. The results showed that the relative luciferase activity decreased obviously in WT after silencing METTL3, and the same change occurred in Mut1, Mut2 and Mut5. However, the luciferase activity of the control group decreased in Mut3 and Mut4 compared with WT, and luciferase activity had no significant change when we silenced METTL3, demonstrating that the most important binding sites were site3 and site4 (Figure 6M).

### 3.8. m^6^A/HNF1A-AS1/CCND1 Axis Cooperated with PDCD4 to Regulate PI3K/AKT Pathway and Related Clinical Relationship in CRC

As a classical oncogene, CCND1 (cyclinD1) regulates the cell cycle to promote cancer progression and its role in colorectal cancer had been demonstrated in several studies. As a typical tumor suppressor, PDCD4 (programed cell death 4) promotes cell apoptosis and inhibits cell cycle through blocking the PI3K/AKT pathway, ultimately affecting the downstream genes, such as cyclinD1 and c-MYC. Thus, we investigated the influence of HNF1A-AS1 on the PI3K/AKT pathway. Western blot results suggested that silencing HNF1A-AS1 suppressed the activation of the PI3K/AKT pathway. CCND1 over-expression or PDCD4 knockdown rescued the inhibition mediated by HNF1A-AS1 knockdown (Figure S3G). The above results suggested that the influence of HNF1A-AS1 on the PI3K/AKT pathway was partly mediated by CCND1 and PDCD4. CCND1 acted as the downstream of PDCD4, indicating that PDCD4 could directly activate the PI3K/AKT pathway to regulate CCND1.

**Figure 7:**
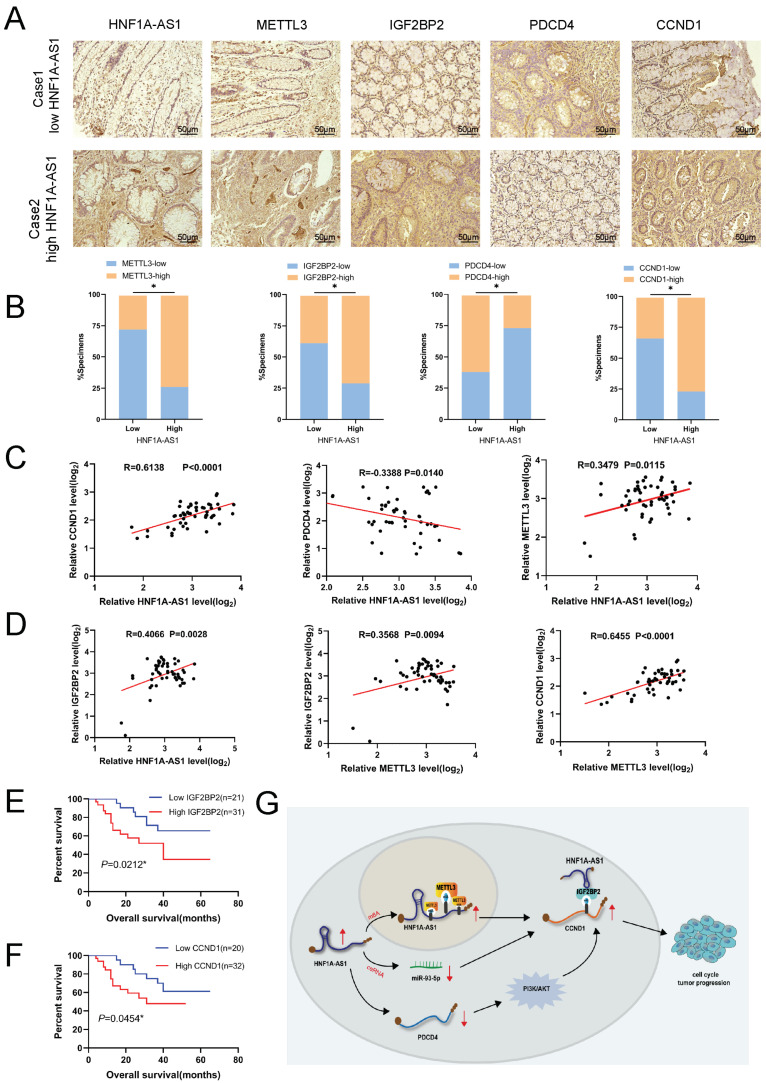
m^6^A/HNF1A-AS1/CCND1 axis cooperated with PDCD4 to regulate PI3K/AKT pathway and related clinical relationship in CRC. (**A**) Representative images showing high or low expression of HNF1A-AS1, METTL3, IGF2BP2, PDCD4 and CCND1 in CRC patients’ tumor tissues. Scar bar: 50 μm. (**B**) Percentages of specimens showing different levels of METTL3, IGF2BP2, PDCD4 and CCND1 in the high or low HNF1A-AS1 expression groups (*n* = 52, Chi-square test). (**C**,**D**) Correlation analysis between HNF1A-AS1, METTL3, CCND1, PDCD4 and IGF2BP2 in our CRC tissues. (**E**) Kaplan–Meier analysis of the patient overall survival based on IGF2BP2 levels in our colorectal cancer patients (log-rank test). (**F**) Kaplan–Meier analysis of the overall survival curves for CRC patients with high expression of CCND1 or low expression of CCND1 (log-rank test). (**G**) The regulatory mechanism of HNF1A-AS1 in promoting CRC proliferation and migration. METTL3-induced m^6^A modification contributed to HNF1A-AS1 upregulation in IGF2BP2-depentent way to promote CCND1 mRNA stability. Meanwhile, HNF1A-AS1 sponged miR-93-5p to promote CCND1 expression and suppressed PDCD4 to upregulate CCND1 by activating PI3K/AKT pathway. * *p* < 0.05.

Next, we analyzed our 52 CRC samples to further investigate the clinical relationship of the HNF1A-AS1–IGF2BP2–CCND1 axis in CRC progression. IHC and ISH assay results (Figure 7A) demonstrated that HNF1A-AS1-high patients were consistent, with high expression of METTL3, IGF2BP2, CCND1 and low expression of PDCD4, while HNF1A-AS1-low patients had the opposite outcomes (Figure 7B). Moreover, RT-qPCR analysis showed that HNF1A-AS1 had a positive correlation with METTL3, IGF2BP2, CCND1 and a negative relation with PDCD4 (Figure 7C and Figure S4A). METTL3 had a positive relation with IGF2BP2 and CCND1 (Figure 7D and Figure S4B). Meanwhile, IGF2BP2 was positively related with CCND1, and PDCD4 was negatively related with CCND1 both in our CRC samples and the TCGA database analysis (Figure S4C,D). Higher expression of IGF2BP2 or CCND1 was associated with unfavorable prognosis for CRC patients (Figure 7E,F). In a nutshell, METTL3-mediated m^6^A modification was attributed to the upregulation of HNF1A-AS1 in a IGF2BP2-dependent way, and HNF1A-AS1 modulated the cell cycle through multiple ways. Apart from suppressing PDCD4 or competitively sponging miR-93-5p, the HNF1A-AS1/IGF2BP2/CCND1 complex further stabilized CCND1 mRNA to promote cell cycle progression (Figure 7G).

## 4. Discussion 

Colorectal cancer (CRC) has become a serious challenge for human life, and its morbidity and mortality in China have been on the rise in recent years. Currently, chemotherapy combined with targeted treatment has become the main treatment for metastatic colorectal cancer. However, the median survival time for CRC patients has not been obviously extended. Therefore, exploring new biomarkers is urgently needed for the diagnosis and prognosis of CRC. Long non-coding RNAs, once defined as having no biological function in human life, have been studied extensively in cancers and regulated colorectal cancer progression. However, the exact mechanisms of long non-coding RNAs are still not very clear. In our research, by searching the TCGA database, we filtered the differentially expressed lncRNA HNF1A-AS1 in colorectal cancer, which was closely related with overall survival. Vivo and vitro experiments were carried out to confirm its biological role in colorectal cancer. Then, we used bioinformatics analysis and screened out CCND1 and PDCD4 as the downstream targets.

CCND1 (CyclinD1) is an important regulatory protein for cell cycle, which can modulate cell cycle transition from the G1 to the S phase. Several studies have demonstrated that CCND1 is positively associated with progression of various tumors [22,23]. In CRC, the over-expression rate of CCND1 reaches 72%, and the expression level of CyclinD1 is related with poor prognosis [24]. The most widely studied mechanism of long non-coding RNAs was competitively sponging miRNAs to affect downstream genes. In our research, CyclinD1 was recognized as the direct target of miR-93-5p. The role of miR-93-5p had been studied in multiple malignancies, such as NCSLC, pancreatic ductal adenocarcinoma, ovarian carcinoma and so on [25,26,27,28,29]. It had been reported that miR-93-5p could downregulate FOXA1 and upregulate TGFB3 to confer radioresistance in CRC. It was also reported that miR-93-5p suppressed CRC progression via targeting PDL-1, and long non-coding RNA CTBP1-AS2 could modulate the miR-93-5P/TGF-beta/SMAD2/3 pathway in colorectal cancer [30,31]. HNF1A-AS1 had a negative relation with miR-93-5p in our clinical tumor tissues, and there were potential binding sites between them from the predicted website. Our further luciferase activity results confirmed the direct combination between CCND1 and miR-93-5p. However, there may be many other miRNAs interacting with HNF1A-AS1 and CCND1 to play biological roles, which required us to further study.

N6-methyladenosine (m^6^A) modification, which occurred in nearly 90% of human mRNAs and ncRNAs at the post-transcriptional level, played biological roles during tumor initiation or progression [32,33]. METTL3 had been reported to promote CRC development through modulating the m^6^A–GLUT1–mTORC1 axis and the m^6^A–CRB3–Hippo axis [34,35]. Wang et al. found that METTL14 mediated by TCF4 and HuR suppressed colorectal cancer progression by silencing ARRDC4 in an m^6^A manner [36]. Similarly, our study demonstrated the function of m^6^A-modified lncRNA HNF1A-AS1 in colorectal cancer. Our result showed that m^6^A modification mediated the upregulation of HNF1A-AS1. Bioinformatic prediction and the MeRIP assay demonstrated m^6^A modification in HNF1A-AS1. During the dynamic m^6^A modification progress, METTL3 acted as an m^6^A writer, and HNF1A-AS1 was recognized by IGF2BP2, a member of the IGF2BPs family, which functioned to stabilize RNA stability. The study by Lang et al. suggested that METTL3-modified lncRNA PCAT6 promoted bone metastasis in prostate cancer via forming the IGF2BP2–IFG1R complex to stabilize IGF1R mRNA [37]. The study by Wu et al. suggested that lncRNA LINRIS promoted glycolysis in CRC by stabilizing IGF2BP2 [38]. Li et al. revealed that METTL3 maintained SOX2 expression in an m^6^A-IGF2BP2-dependent manner to facilitate CRC progression [39]. Our research revealed that the half-time of HNF1A-AS1 was significantly decreased after silencing METTL3 in an m^6^A-dependent way, and HNF1A-AS1 regulated the CCND1 mRNA stability through binding IGF2BP2 in an m^6^A-dependent way. Apart from affecting lncRNA attenuation, m^6^A modification could influence the interaction of lncRNA and RNA binding protein (RBP). Whether the secondary structure of HNF1A-AS1 was influenced by m^6^A modification was still unclear, and we would further investigate the structure change between the HNF1A-AS1 and IGF2BP2.

As a classical tumor suppressor in multiple cancers, a recent study has revealed a certain regulatory relationship between PDCD4 and CCND1. PDCD4 could hinder the PI3K/AKT pathway and the downstream factors CCND1 and c-MYC to suppress the cell cycle. PDCD4 can regulate the expression of miR-374a by inhibiting the PI3K/AKT/c-JUN signaling pathway, thereby affecting the expression of CCND1 [40]. Our results are consistent with such regulatory relationship. HNF1A-AS1 suppressed the expression of PDCD4 to activate the PI3K/AKT pathway and ultimately promoted CCND1 expression to accelerate cell proliferation. However, whether HNF1A-AS1 could regulate PDCD4 through m^6^A modification is unclear. HNF1A-AS1 could interact with YTHDFs and YTHDCs family proteins as well in our predicted results from the RPISeq website. At the same time, PDCD4 mRNA had several potential m^6^A-modified sites from the SRAMP website. The precise molecular mechanism of their interaction needs further exploration.

Long non-coding RNAs have been widely studied in multiple malignancies and have affected tumor progression. However, the definite biological mechanisms behind its dysregulation are still being researched. HNF1A-AS1 was first discovered and observed to be upregulated in human EACs. Silencing HNF1A-AS1 blocked the cell cycle and suppressed cell proliferation, which was partly mediated by chromatin and nucleosome assembly [41]. It was also reported that HNF1A-AS1 affected NSCLC radiosensitivity via competitively sponging miR-92a-3p and ultimately regulating the JNK pathway [42]. HNF1A-AS1 was studied extensively ingastrointestinal carcinomas, such as hepatocellular carcinoma, gastric cancer, colorectal cancer and so on. In gastric cancer, HNF1A-AS1 induced by EGR1 was shown to promote the cell cycle as well. Apart from EGR1, HNF1α could regulate the transcription of HNF1A-AS1 as well. HNF1A-AS1 activated SHP-1 via phosphorylation, demonstrating that the HNF1α/HNF1A-AS1/SHP-1 axis may become a new treatment in hepatocellular carcinoma [43]. Fang et al. reported its function in colon cancer, and HNF1A-AS1 suppressed the miR-34a/SIRT1/p53 feedback loop to facilitate tumor metastasis [20]. These studies revealed a correlation between HNF1A-AS1 and CCND1. However, it has not been elucidated clearly how HNF1A-AS1 regulates CCND1 to modulate the cell cycle. In our study, our results showed that HNF1A-AS1 could interact with IGF2BP2 to stabilize CCND1 mRNA by m^6^A modification. On the other hand, it could regulate CCND1 through competitively sponging miR-93-5p. It also suppressed PDCD4 to activate the PI3K/AKT pathway and ultimately activate CCND1 to accelerate the cell cycle. However, the silver staining and mass spectrometry demonstrated that there still exist other RNA binding proteins (RBPs), which could interact with HNF1A-AS1 and mediate the HNF1A-AS1 function. We will carry out further studies to investigate the relationship between these RBPs and HNF1A-AS1. Our results illustrated multiple mechanisms of HNF1A-AS1 modulating the cell cycle, showing that HNF1A-AS1 had the potential to become a biomarker in colorectal cancer prognosis.

## 5. Conclusions

In a nutshell, our study revealed that HNF1A-AS1 regulates the cell cycle in several ways. m^6^A-modified HNF1A-AS1 interacted with IGF2BP2 to stabilize CCND1 mRNA. HNF1A-AS1 could also upregulate CCND1 by sponging miR-93-5p or suppressing PDCD4 to promote cell cycle progression. Our study indicated that HNF1A-AS1 has the potential in CRC prognosis and could serve as a biomarker in colorectal cancer prognosis.

## Figures and Tables

**Table 1:** Correlation between HNF1A-AS1 expression and clinicopathological characteristics of 52 CRC patients.

Variables	Total Patients	Low Expression of HNF1A-AS1	High Expression of HNF1A-AS1	*p* Value Chi-Squared Test
All cases	52	18	34	
Age ≤60 >60	20 32	8 10	12 22	0.5188
Sex male female	30 22	11 7	19 15	0.7165
TNM stage I and II III and IV	27 25	13 5	14 20	0.0330 *
Tumor size ≤4 cm >4 cm	24 28	13 5	11 23	0.0061 **
Differentiation High/moderate Poor	44 8	15 3	29 5	0.8278
Lymph node N0 N1/2/3	27 25	13 5	14 20	0.0330 *
Distant metastasis Negative Positive	37 15	16 2	21 13	0.0400 *

* *p* < 0.05, ** *p* < 0.01.

## Data Availability

Please contact the corresponding authors for all data requests.

## Supplementary Materials

The following supporting information can be downloaded at: https://www.mdpi.com/article/10.3390/cells11193008/s1, Figure S1: Additional functional assays of HNF1A-AS1 in vitro; Figure S2: Additional rescue assays in vitro; Figure S3: Additional rescue assays in vitro; Figure S4: Additional correlation analysis; Table S1: Sequences of all the shRNAs; Table S2: Primer sequences for RT-qPCR.

## Abbreviations

lncRNA, long non-coding RNA; CRC, colorectal cancer; FISH, fluorescence in situ hybridization; TCGA, The Cancer Genome Atlas; CCND1, CyclinD1; PDCD4, programed cell death 4; IGF2BP2, insulin-like growth factor 2 mRNA binding protein 2; m^6^A, N6-methyladenosine; H&E, hematoxylin and eosin stain; ISH, in situ hybridization; RIP, RNA immunoprecipitation; IHC, immunohistochemistry; EMT, epithelial-mesenchymal transition
