# Supplementary material for: m6A Modification of Long Non-Coding RNA HNF1A-AS1 Facilitates Cell Cycle Progression in Colorectal Cancer via IGF2BP2-Mediated CCND1 mRNA Stabilization
Source: Cells. 2022 Sep 27;11(19):3008. doi: 10.3390/cells11193008 (PMC9562639; doi:10.3390/cells11193008)
Supplement: Supplementary file 1 [file cells-11-03008-s001.zip › cells-1878386-supplementary.pdf]

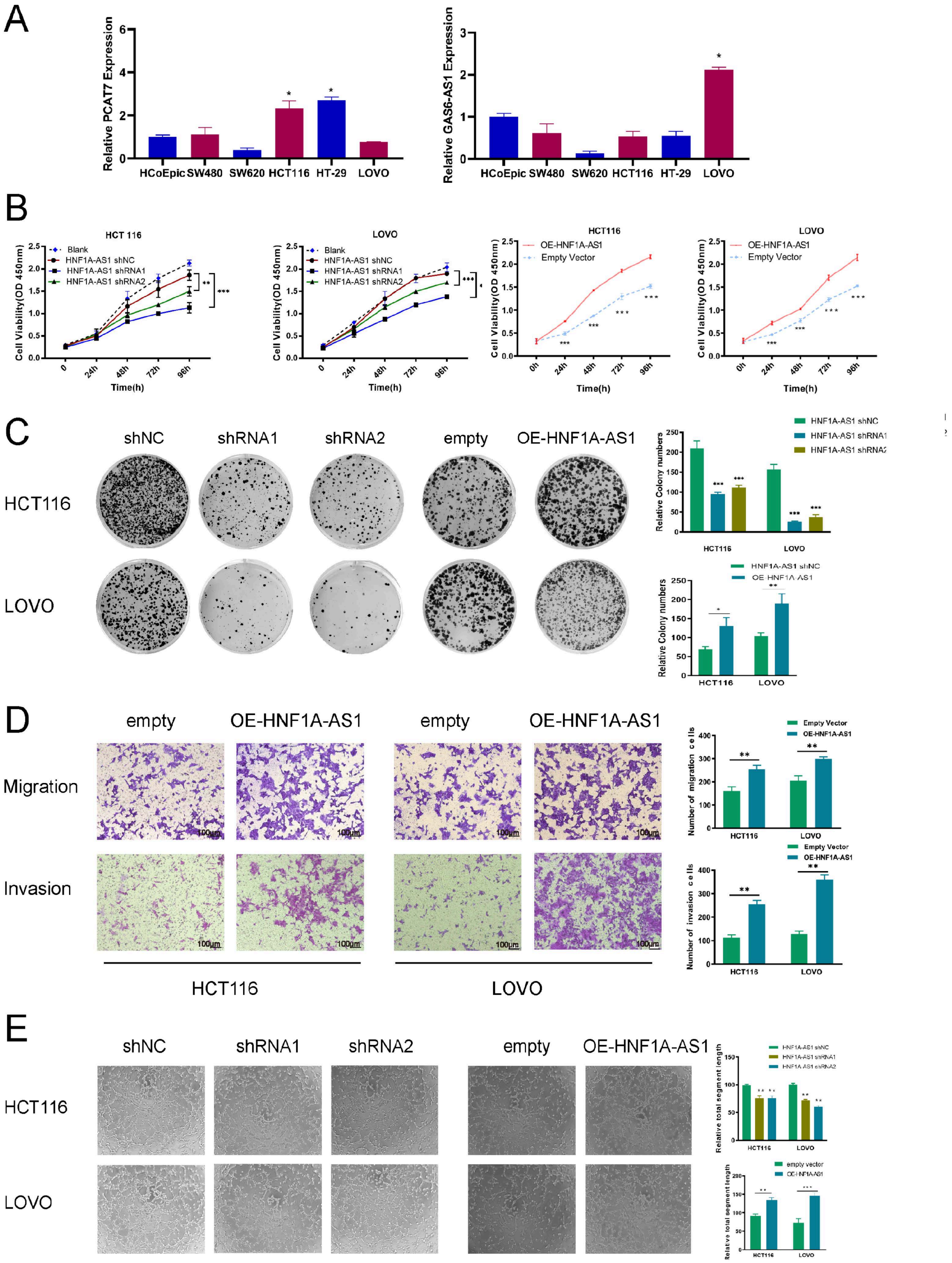

Fig. S1 Additional functional assays of HNF1A-AS1 in vitro.

A. The expression of another two lncRNAs (PCAT7 and GAS6-AS1) was detected in several CRC cell lines through RT-qPCR.

B and C. CCK8 and colony formation assays were performed to detect the proliferation ability of HCT116 and LOVO cells transfected with shHNF1A-AS1 or over-expressing plasmid of HNF1A-AS1.

D. Transwell assays were performed to explore cell migration and invasion abilities when over-expressing HNF1A-AS1.

E. The change of cell angiogenesis ability was detected through tube formation assay after silencing or over-expressing HNF1A-AS1. Statistical analysis was performed through Student's *t*-test. *P<0.05, **P<0.01, ***P<0.001

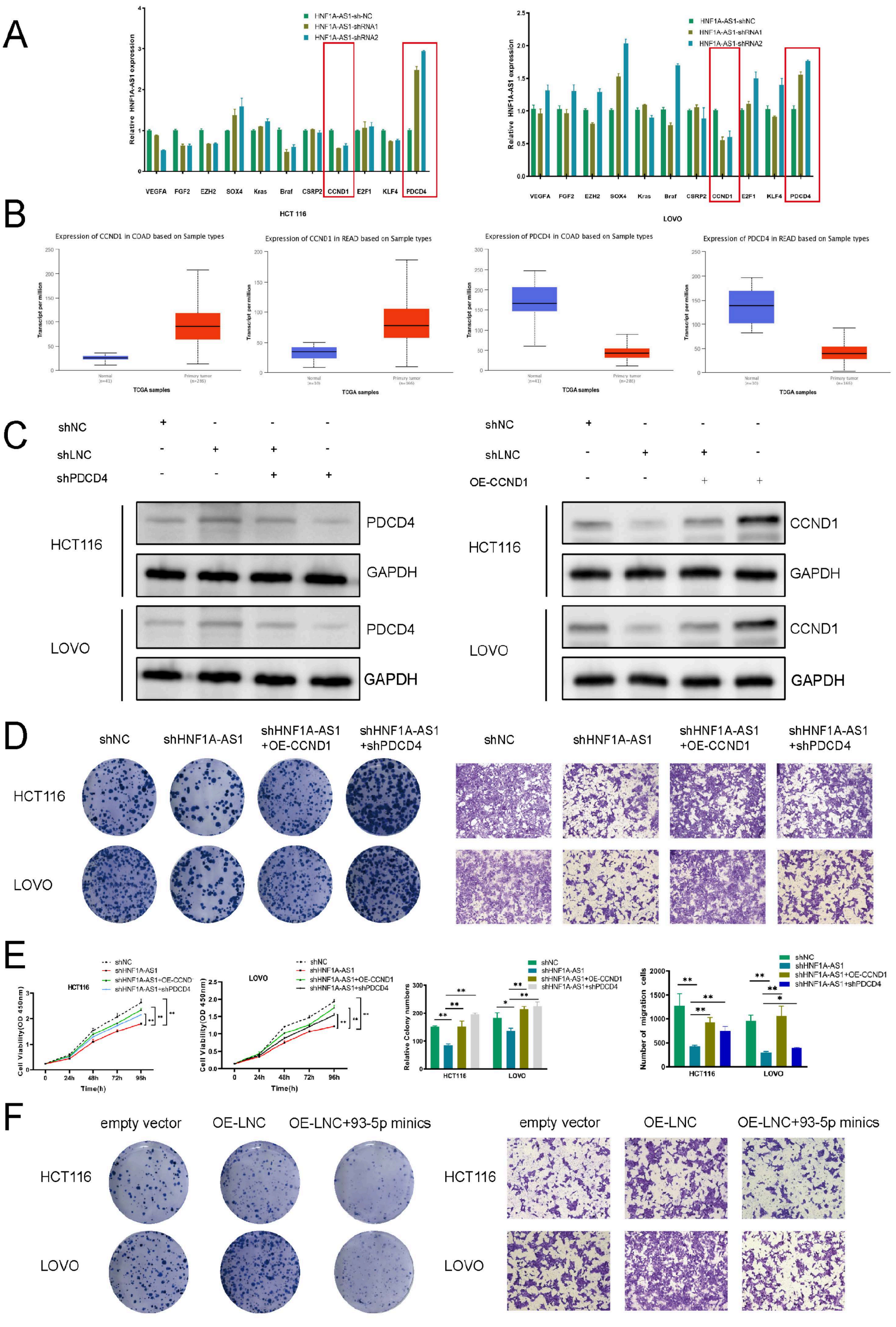

Fig. S2

A. Potential targeted genes were verified in HCT116 and LOVO cells through RT-qPCR.

B. The expression of CCND1 and PDCD4 in COAD and READ from TCGA database.

C. CCND1 and PDCD4 were detected by western blot in functional rescue assays.

D and E. Knockdown of PDCD4 or over-expressing CCND1 reversed the proliferation and migration induced by silencing HNF1A-AS1 through CCK8, colony formation and transwell assays.

F. Over-expressing of miR-93-5p abolished the proliferation and migration induced by over-expressing HNF1A-AS1 through colony formation and transwell assays. Statistical analysis was performed through Student's *t*-test. **P*<0.05, ***P*<0.01

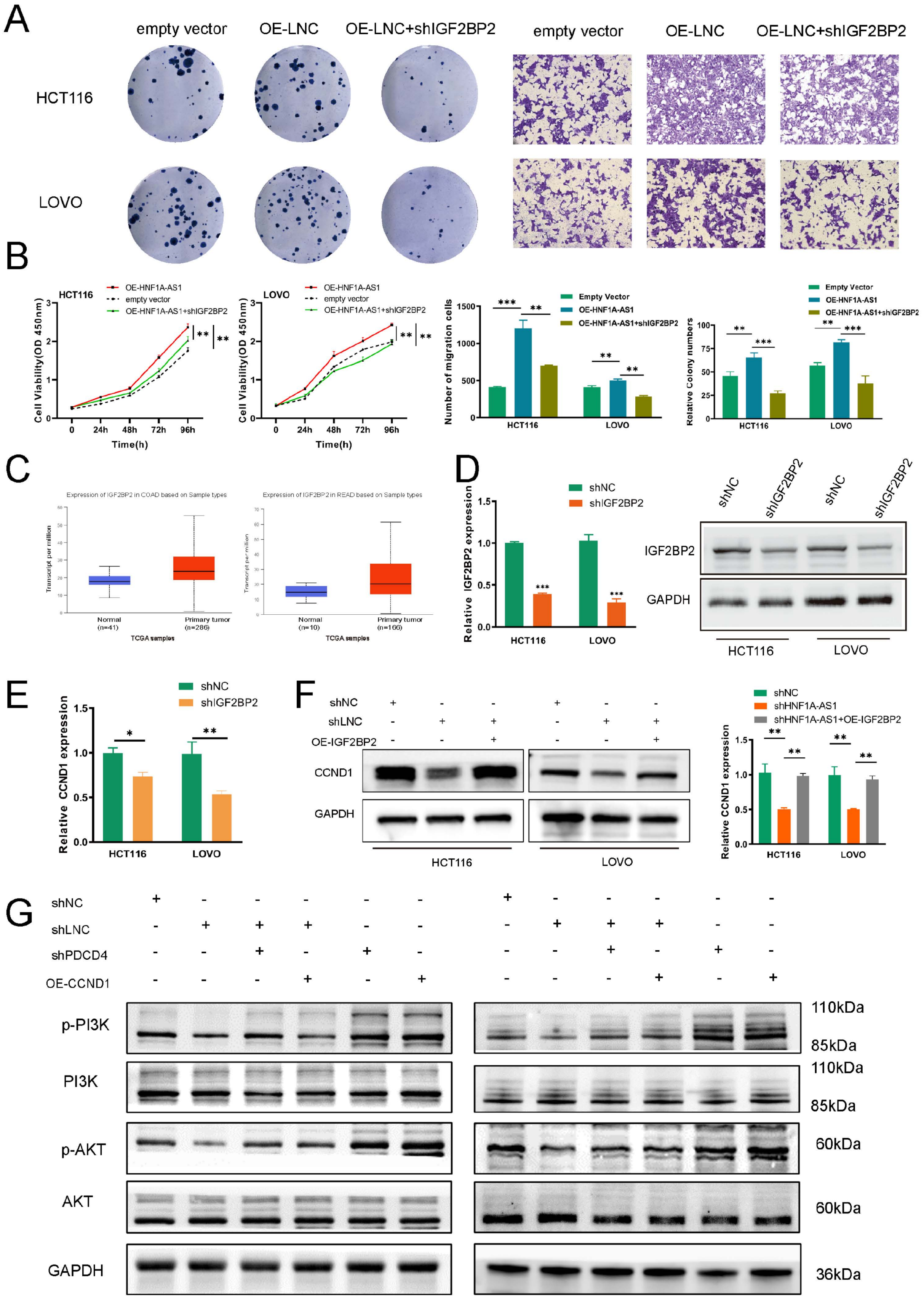

Fig. S3

A and B. Knockdown of IGF2BP2 reversed the proliferation and migration induced by over-expressing HNF1A-AS1 through CCK8, colony formation and transwell assays.

C. The expression of IGF2BP2 in COAD and READ from TCGA database.

D. Knockdown efficiency of IGF2BP2 were verified through RT-qPCR and western blot.

E. The expression of CCND1 was decreased after silencing IGF2BP2 through RT-qPCR.

F. Over-expressing IGF2BP2 reversed the CCND1 mRNA and protein expression induced by silencing HNF1A-AS1.

G. Western blot analysis of AKT, p-AKT, PI3K and p-PI3K expression in rescue assays.

Statistical analysis was performed through Student's *t*-test.

**P*<0.05, ***P*<0.01, ****P*<0.001

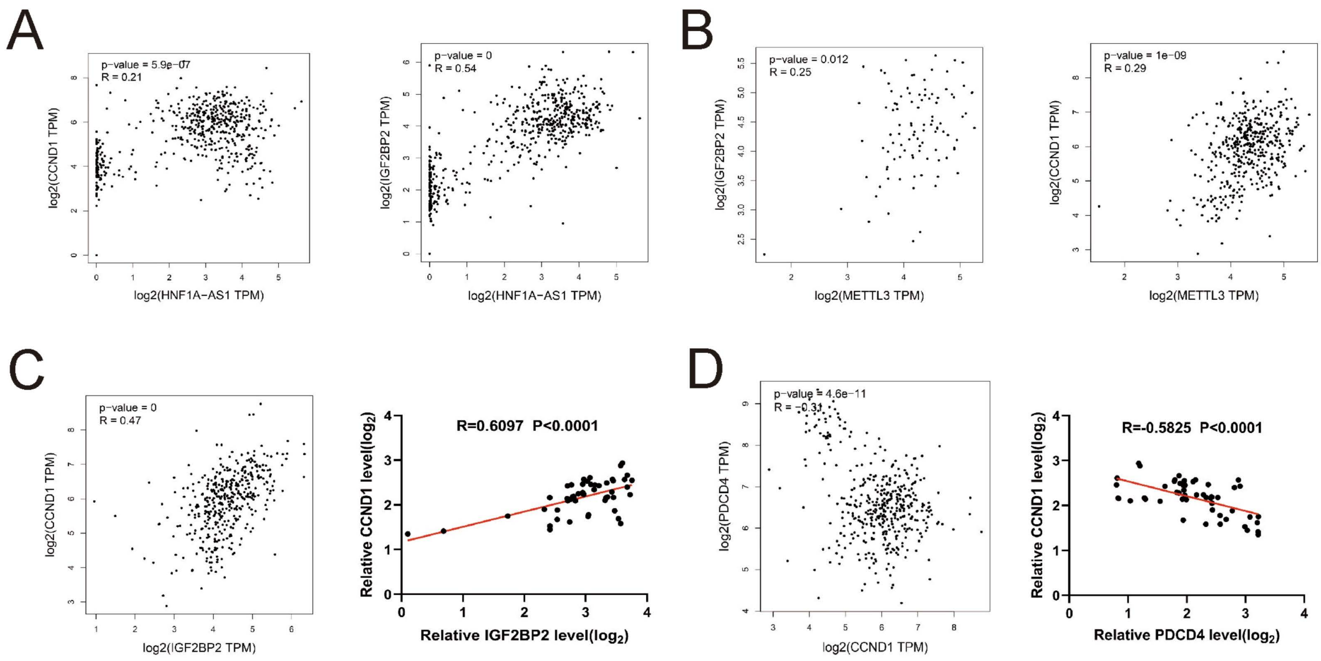

Fig. S4

A. The correlation analysis between HNF1A-S1, CCND1 and IGF2BP2 from TCGA database.

B. The correlation analysis between METTL3, CCND1 and IGF2BP2 from TCGA database.

C. The correlation analysis between IGF2BP2 and CCND1 from TCGA database and

our CRC tissues.

D. The correlation analysis between CCND1 and PDCD4 from TCGA database and our CRC tissues.

Table S1 Sequences of all the shRNA

Gene	Direction	Sequence
miRNA minics NC	sense	CAGUACUUUUGUGUAGUACAA
miR-93-5p minics	sense	CAAAGUGCUGUUCGUGCAGGUAG
	antisense	ACCUGCACGAACAGCACUUUGUU
sh-IGF2BP2	sense	CAUGCCGCAUGAUUCUUGATT
	antisense	UCAAGAAUCAUGCGGCAUGTT
sh-PDCD4	sense	GCAUGGAGAUACUAAUGAATT
	antisense	UUCAUUAGUAUCUCCAUGCTT
sh-HNF1A-AS1-1	sense	CACCUGCAUUCAAACUCGGACUGUU
	antisense	AACAGUCCGAGUUUGAAUGCAGGUG
sh-HNF1A-AS1-2	sense	CCCUCCAUCUAACAUUCAATT
	antisense	UUGAAUGUUAGAUGGAGGGTT
sh-HNF1A-AS1-3	sense	GGGUGAGCAGCUGUUUGCAAGACUA
	antisense	UAGUCUUGCAAACAGCUGCUCACCC
sh-METTL3	sense	GAC UGC UCU UUC CUU AAU ATT
	antisense	UAU UAA GGA AAG AGC AGU CTT
sh-METTL14	sense	GGA AGA GUG UGU UUA CGA ATT
	antisense	UUC GUA AAC ACA CUC UUC CTT

Table S2 Primer sequences for RT-qPCR

Gene	5'-3'	3'-5'
HNF1A-AS1	AGTCGAGAGCGTGGGAAAC	AACATACTGGCCCAAACAGC
GAPDH	GAAGGTGAAGGTCGGAGT	GAAGATGGTGATGGGATTTC
U6	CTCGCTTCGGCAGCACA	AACGCTTCACGAATTTGCGT
VEGFA	CTACCTCCACCATGCCAAGT	CACACAGGATGGCTTGAAGA
FGF2	AAGACCAACCTGGTGAAACC	TCACTGCAACCTTGACCTCTC
EZH2	TAGGGAAGCAGGGACTGAAA	CTTCAGCACCACTCCACTCC
SOX4	TCGCTGTCGGGTCTCTAGTT	ACTGTGGCTCCAGGACTCTC
Kras	TGTGGTAGTTGGAGCTGGTG	CATGTACTGGTCCCTCATTGC
Braf	CATGGGCTATTCCACAAAGC	AAGTAATCCATGCCCTGTGC
CSRP2	ACAACAGTGGCAATTCACGA	TGAGGCTGAACACTCTCTGG
CCND1	ATGCTGGGCACTTCATCTG	TGGTGGCACGTAAGACACAC
E2F1	CATCAGTACCTGGCCGAGAG	TCAAGGACGTTGGTGATGTC
KLF4	GGCCACCGGACCTACTTACT	GGCCGAGATCCTTCTTCTTT
PDCD4	TCAGCATCCTCCATTAACGA	ATCGCCTATCCAGCAACCTT
miR-93-5p	CGCAAAGTGCTGTTCGTGC	AGTGCAGGGTCCGAGGTATT
METTL3	GCTGACCATTCCAAGCTCTC	ATTTCTTGGCTGGCTCCTTT
IGF2BP2	TTGCAGGAATTGACGCTGTA	ACCCAAGGCGTTCAGATTTA
